# Various therapies for ocular surface diseases

**Published:** 2018

**Authors:** Alina Gheorghe, Ancuţa Teodora Rosoga, Fildys Mrini, Iulia Vărgău, Florentina Gherghiceanu

**Affiliations:** * Clinical Emergency Eye Hospital, Bucharest, Romania; ** Sanamed Hospital, Bucharest, Romania; *** ”Carol Davila” University of Medicine and Pharmacy, Bucharest, Romania

**Keywords:** cornea, optical coherence tomography, cross-linking, amniotic membrane

## Abstract

This paper aims to discuss various therapeutical strategies (corneal cross-linking, amniotic membrane transplantation with or without autologous serum application, medical regenerative therapy) for treating ocular surface diseases according to medical indications, etiology, local and general status of the patient. Besides the evolution and treatment of the lesions induced by corneal foreign body, ocular burns, neurotrophic keratitis, pterygium removal, Mooren’s ulcer, this paper also follows and evaluates the migration, stratification and development of corneal epithelium. Of course, the success of the treatment depended on the therapeutic approach, the cause of the disease, the status of the eye and the patients’ compliance. All cases presented had good results, proving once again, that a well-chosen therapeutic approach ensures the improvement or cure for many ocular surface diseases nowadays.

Abbreviations: BVCA = best corrected visual acuity, OCT = optical coherence tomography, CLX = corneal cross-linking

## Introduction

Being the first and most important refractive component of the eye, the cornea is essential for human vision. Regardless of the etiology, traumatic, infectious, degenerative, or secondary to a systemic disease, ocular surface lesions affect vision either directly or due to complications. Repair and restoration of damaged cornea is a current topic in ophthalmology but also in regenerative medicine and tissue engineering. Successful treatment depends on the lesion, etiology, presence of limbal stem cells status or other complications. Therefore, a judicious therapy from the beginning of the disease or according to its complications underlies a successful treatment.

## Case report

We present three cases of different etiologies that underwent different types of treatment according to pathology, medical eye history, stage of the disease.

**Fig. 1:**
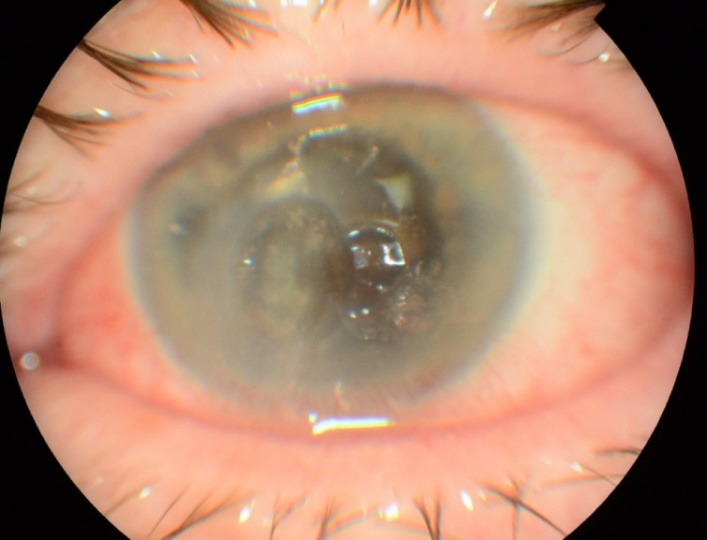
Non-healing corneal ulcer

The first case was of a single eye of a 63-year-old male patient who had a non-healing corneal ulcer with corneal melting developed in the last 3 months. BCVA was 0.1 (Snellen chart) **Fig. 1**. 

**Fig. 2:**
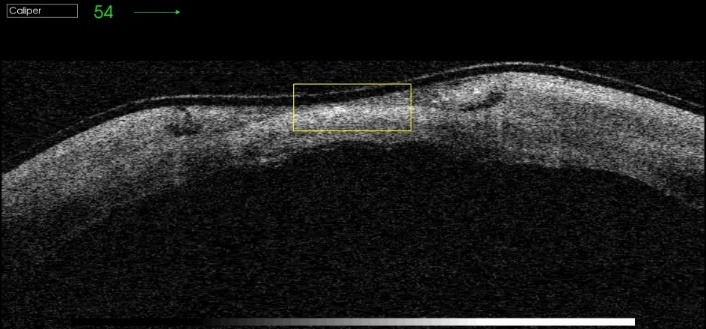
Therapeutic contact lens on the corneal defect

**Fig. 3:**
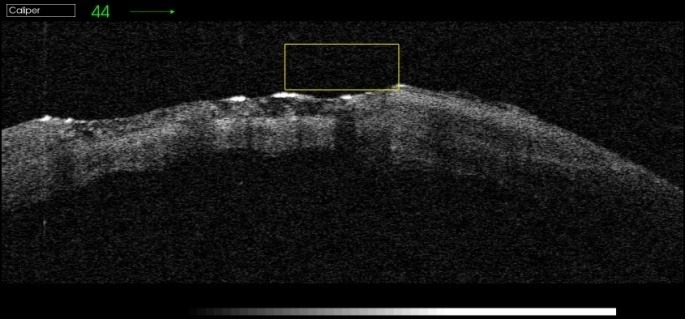
Removal of therapeutic contact lens

**Fig. 4:**
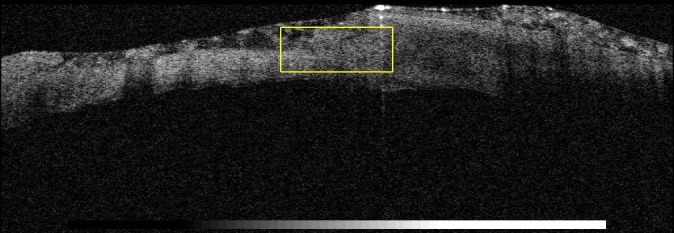
Fragile and unstable epithelium

The failure of proper and complete re-epithelialization of the corneal epithelium was highlighted by the OCT images 1 day after the removal of the therapeutical corneal contact lens. The images show a fragile and unstable epithelium (**Fig. 2**-**4**).

**Fig. 5:**
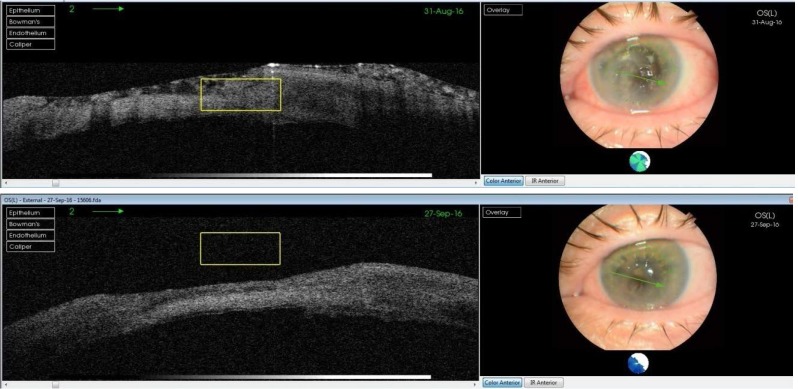
Pre and post CLX intervention

For this case, we chose to perform corneal cross-linking. One month after the procedure, the corneal epithelium was well organized and stable without tendency to break (**Fig. 5**).

**Fig. 6:**
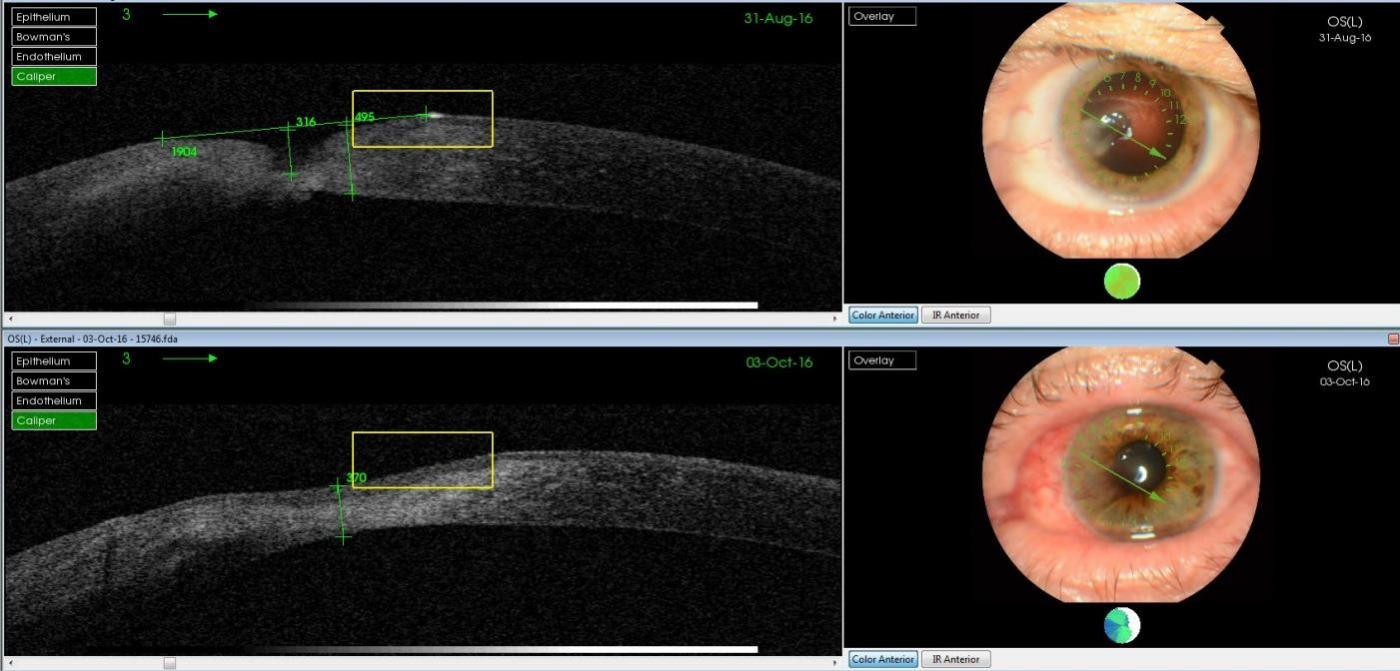
Large corneal defect after foreign body removal treated with amniotic membrane; pre and postoperatory images

The second case was of a male who had a large corneal defect 1 month after the removal of an intracorneal foreign body. BCVA was 20/ 20 (Snellen chart) **Fig. 6** SLD7. The OCT image (**Fig. 6**) showed a defect of 316 microns and a perilesional corneal thickness of 495 microns. Amniotic membrane surgery was performed after all the medical therapeutic options were tried. In this case, the amniotic membrane acted as a scaffold for host cells to populate, thus facilitating healing and repair. It also acted as a space filler to increase stromal thickness [**1**]. Five weeks after the surgery, the corneal defect healed and a normal corneal architecture was achieved (**Fig. 6**).

**Fig. 7:**
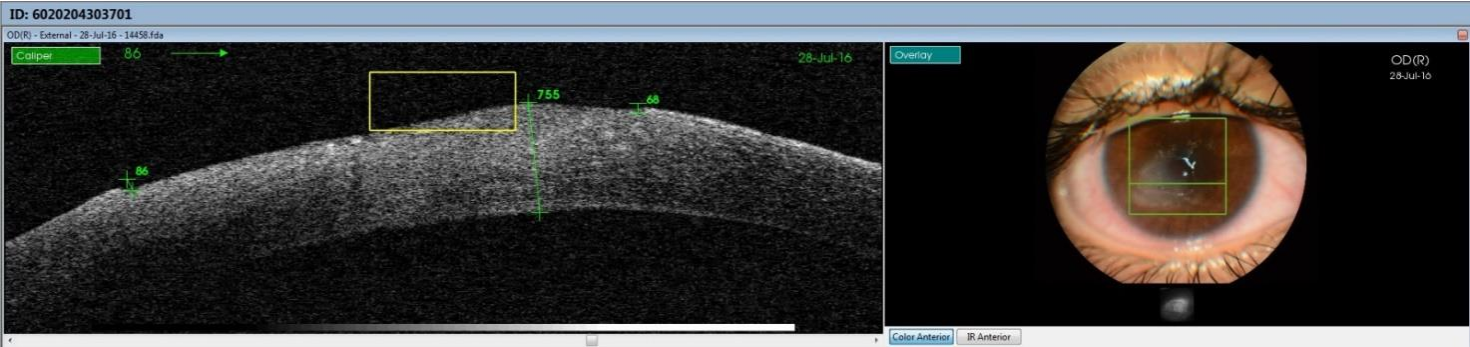
Large epithelial defect and corneal edema

The last case to be presented was of a 14-year-old female with neurotrophic keratitis and corneal ulcer after Bell’s palsy and surgery for venous malformation of cerebral vessels. BCVA was 0.05 (Snellen chart). OCT images showed large epithelial defect and corneal edema ranging from 642 microns to 755 microns (**Fig. 7**).

**Fig. 8:**
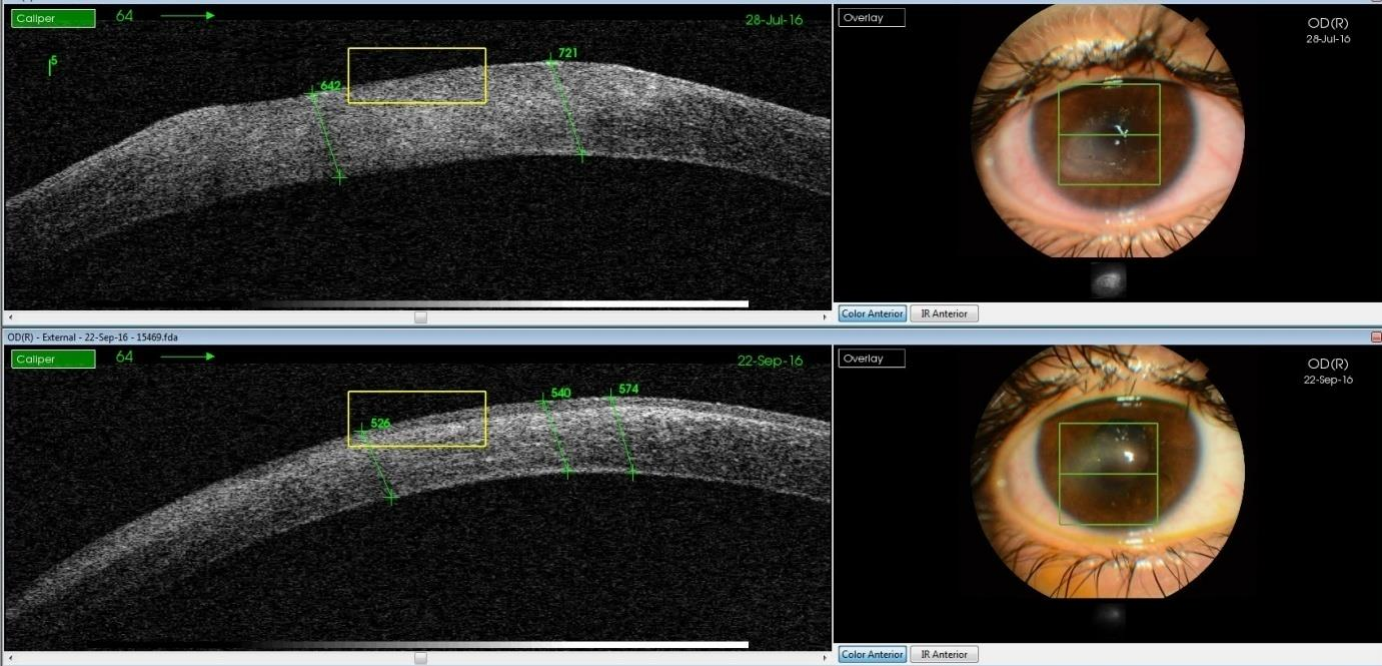
Complete epithelization and reduction of corneal edema from approx. 700 microns to 520 microns

**Fig. 9:**
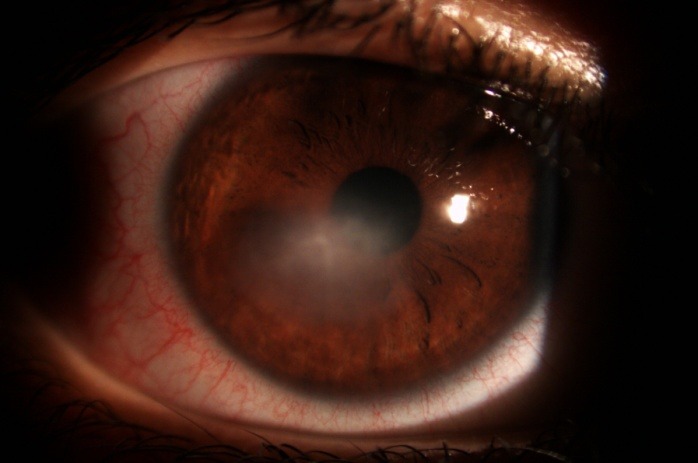
Slit lamp image of the healed neurotrophic keratitis

Together with medical therapy (hyaluronic acid, vitamins, carboxymethyl glucose sulfate, hypromellose) surgical therapy with amniotic membrane graft assured a complete corneal epithelization and remission of corneal edema (**Fig. 8**,**9**).

## Discussion

Ocular surface disorders represent a broad spectrum of conditions. Thanks to the advances in this pathology, treatment options have improved in the last decade. Various methods of ocular surface healing and reconstruction are nowadays available for patients, giving them a real chance of minimizing their visual loss [**2**-**4**].

Regarding our first patient, the causes and mechanisms of his non-healing corneal ulcer were complex. Perhaps a neurotrophic cause, limbal stem cells deficiency, and systemic condition that led to melting cornea phenomenon contributed to his ocular pathology. Cross-linking procedure offered the possibility of intervening on the microstructure of the stromal collagen, increasing the fibril interconnections and microfibrils. After CLX, the corneal cells were more stable and the architecture more durable, preventing corneal perforation.

Corneal epithelium cells renewal follows several theories; the main theory is the one of limbal stem cell that renews the epithelium at every 15 days by cell migration from the limbus to the centre of cornea. Also the normal healing of a corneal erosion or ulcer implies the migration of epithelial basal cells in a monocellular layer that covers the denuded corneal stroma in a centripetal way. Deep corneal ulcers need medical and surgical therapy for healing because the stroma is also affected [**5**].

Amniotic membrane is very conducive to epithelial cell migration and attachment; keratocytes have been shown to re-populate the amnion stroma, thus building corneal stromal tissue. The mechanisms of action of the membrane are attributed to and inferred from its physical structure and its molecular constituents. The amniotic membrane is composed of a single layer of epithelial cells, basement membrane, and avascular stroma. Enzymes, cytokines (IL-6, IL-10), growth factors (EGF, KGF, HGF, and TGF), metalloproteases, and inhibitors of metalloproteases have been identified in amniotic membrane layers [**6**-**9**]. 

## Conclusion

Ocular surface diseases have multiple causes, so a successful therapeutic approach depends not only on the clinical experience but also on the well-chosen therapy. A combined therapy, pharmaceutical and surgical, should not exclude each other, but should be complementary. 

## References

[1] Rahman I, Said DG, Maharajan VS, Dua HS (2009). Amniotic membrane in ophthalmology: indications an limitations. Eye.

[2] McGrath D (2016). Regeneration Revolution, Advances in regenerative medicine leading to paradigm shift in treating ocular surface disorders. Eurotimes.

[3] Gheorghe A, Pop M, Burcea M, Serban M, Zemba M (2016). New clinical application of amniotic membrane transplant for ocular surface disease. Journal of Medicine and Life.

[4] Gheorghe A, Pop M, Mrini F, Vargau I, Barac R (2016). Ocular surface reconstruction in limbal stem cell deficiency. Romanian Journal of Ophthalmology.

[5] Muraine M, Gueudry J, Duchesne B, Majo F (2015). Ulceres chroniques de la Cornee. Laboratoires Thea.

[6] Gheorghe A, Pop M, Tataru CP, Mihai C, Cioboata M (2015). Human amniotic membrane for sever alkali burn-100% visual recovery. Research and Science Today.

[7] Keelan JA, Sato T, Mitchell MD (1997). Interleukin (IL)-6 and IL-8 production by human amnion: regulation by cytokines, growth factors, glucocorticoids, phorbol esters, and bacterial lipopolysaccharide. Biol Reprod.

[8] Koizumi NJ, Inatomi TJ, Sotozono CJ, Fullwood NJ, Quantock AJ, Kinoshita S (2000 ). Growth factor mRNA and protein in preserved human amniotic membrane. Curr Eye Res.

[9] Kubo M, Sonada Y, Muramatsu R, Usui M (2001 ). Immunogenicity of human amniotic membrane in experimental xenotransplantation. Invest Ophthalmol Vis Sci.

